# Supplementary figures and images for: Cryo-EM structure of the CBC-ALYREF complex
Source: eLife. 2024 Sep 16;12:RP91432. doi: 10.7554/eLife.91432 (PMC11405014; doi:10.7554/eLife.91432)

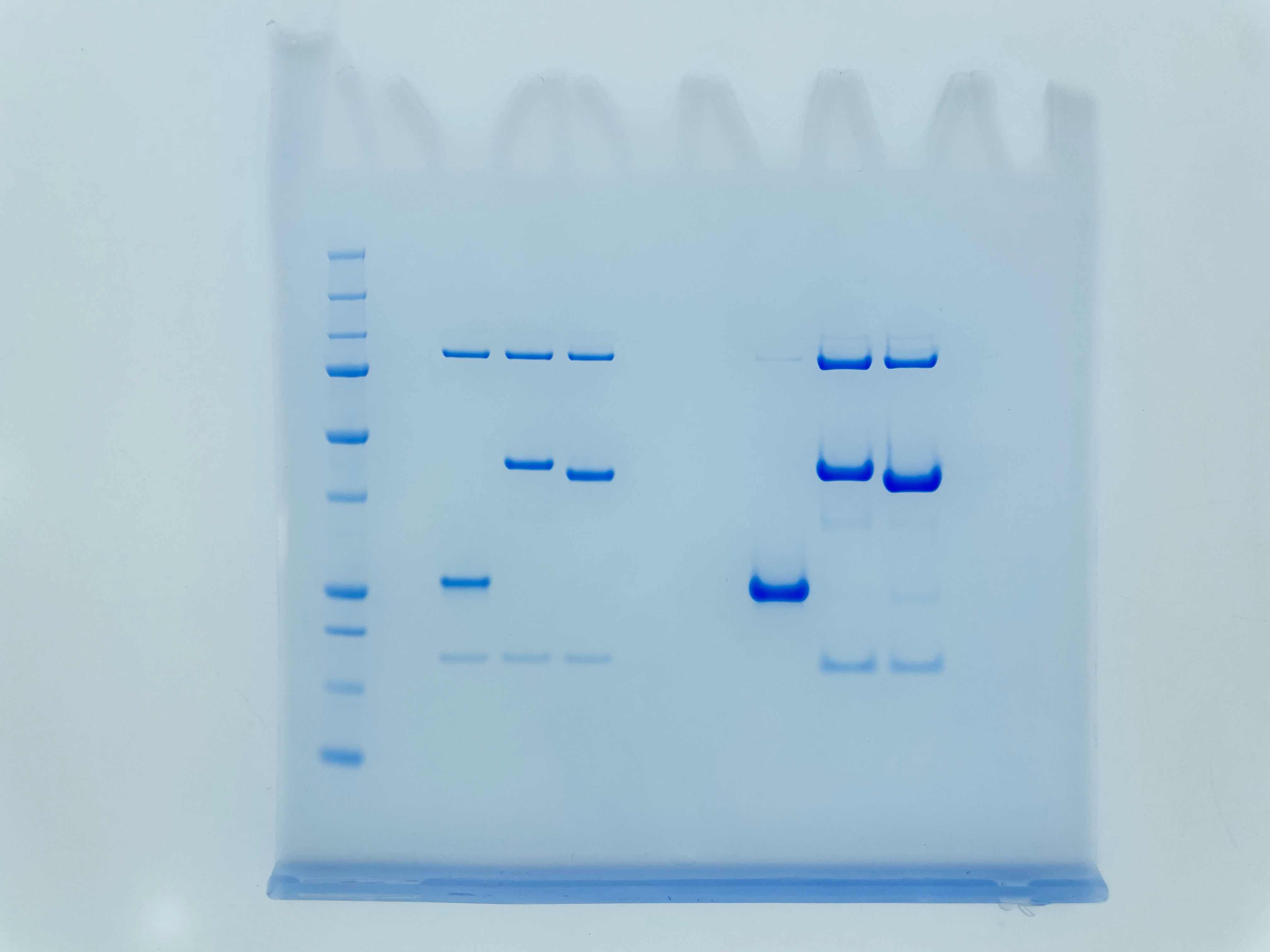

Supplement: Figure 1—source data 1. [file elife-91432-fig1-data1.zip › Figure 1-source data 1/Figure1B_original_gel.png]

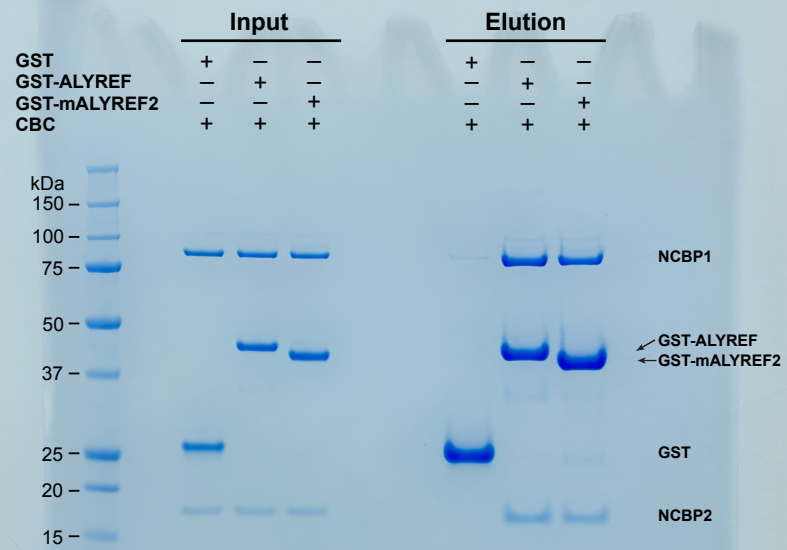

Supplement: Figure 1—source data 1. [file elife-91432-fig1-data1.zip › Figure 1-source data 1/Figure1B_gel_with_label.pdf]

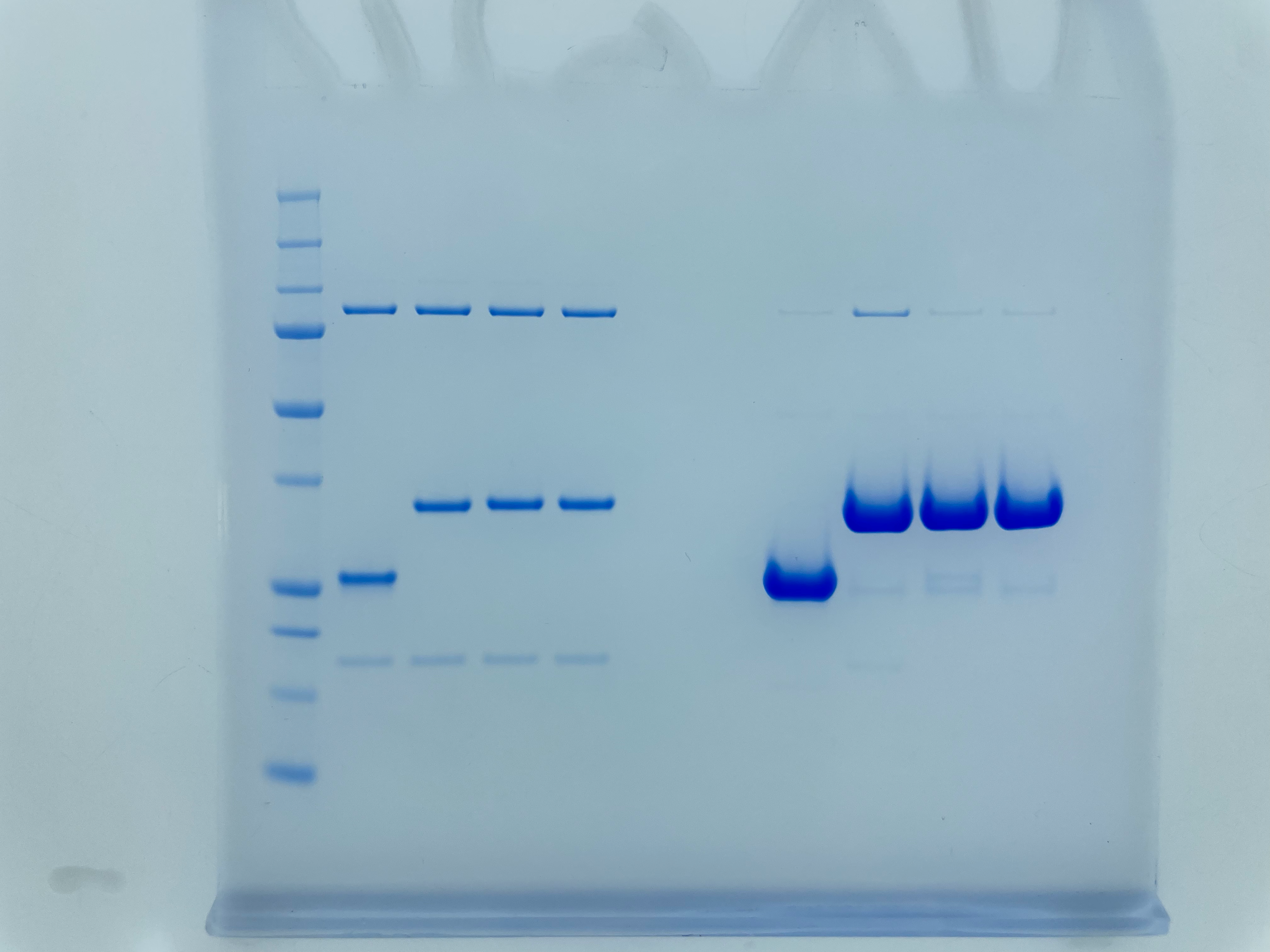

Supplement: Figure 4—source data 1. [file elife-91432-fig4-data1.zip › Figure 4-source data 1/Figure4A_original_gel.png]

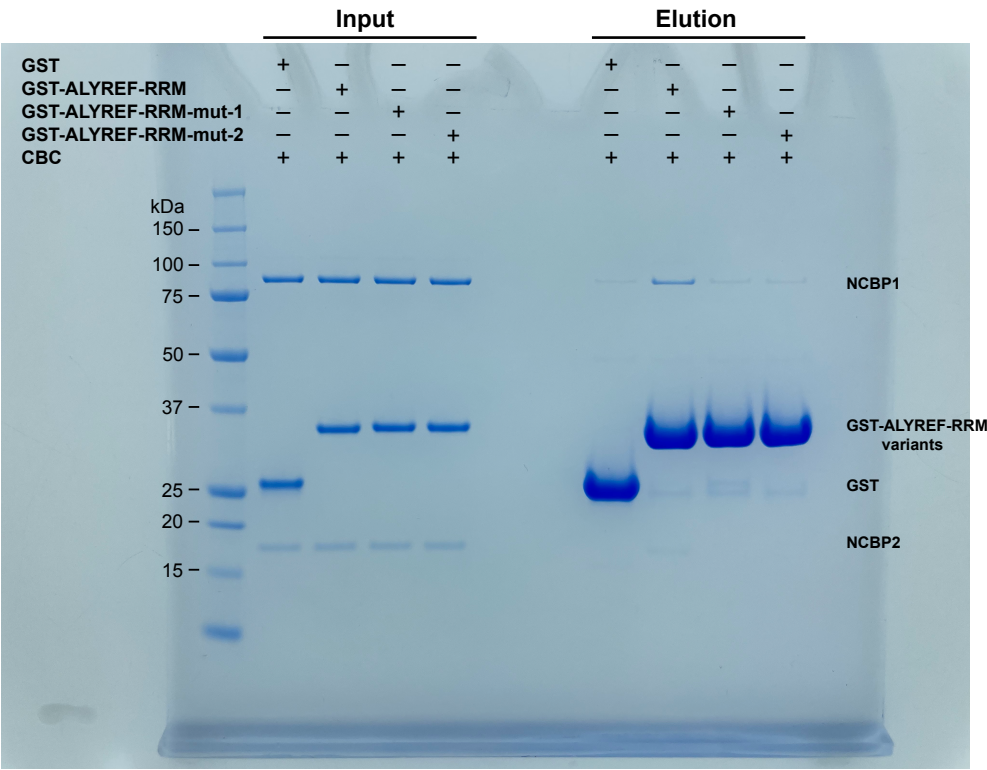

Supplement: Figure 4—source data 1. [file elife-91432-fig4-data1.zip › Figure 4-source data 1/Figure4A_gel_with_label.pdf]

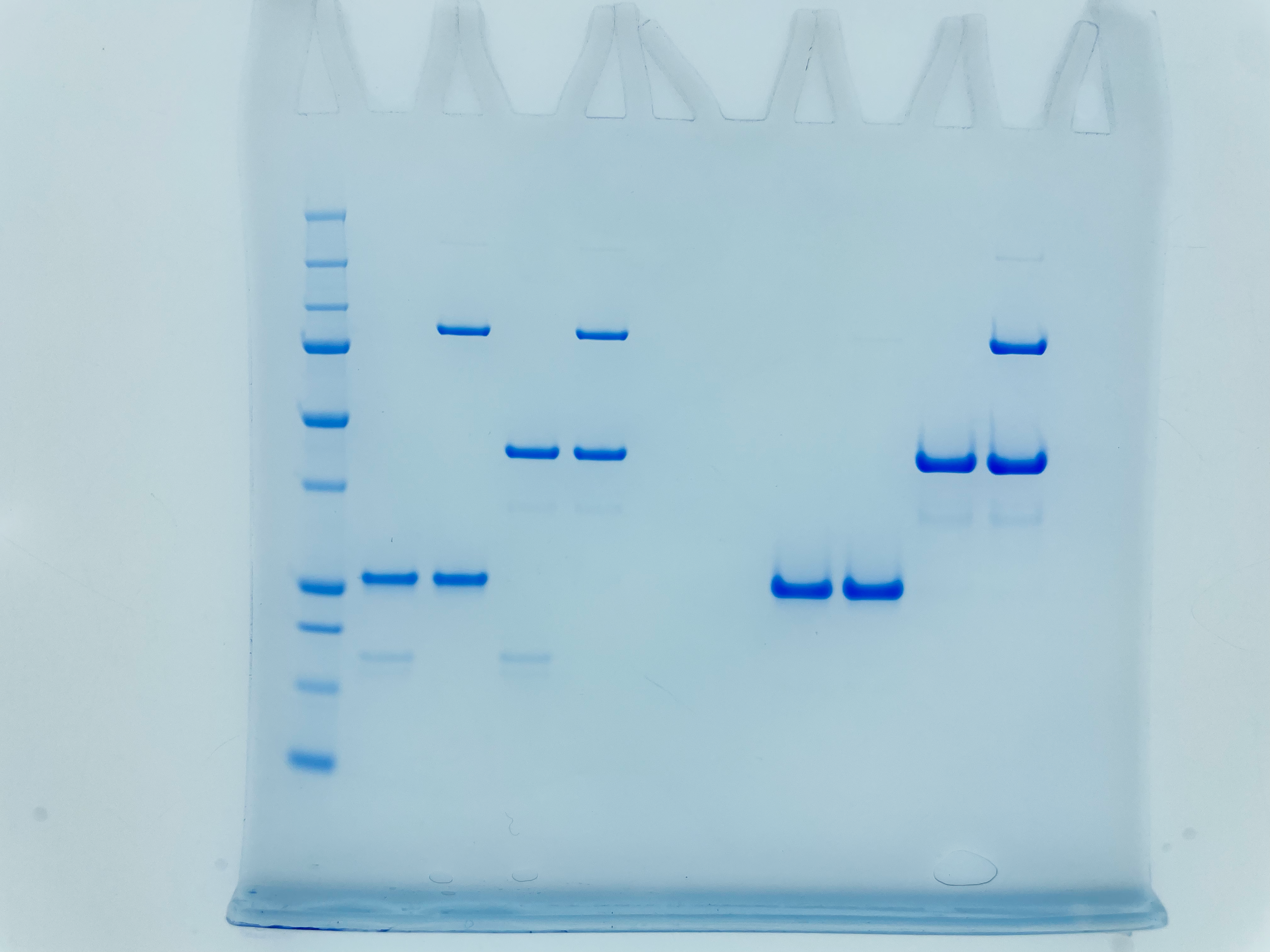

Supplement: Figure 4—source data 2. [file elife-91432-fig4-data2.zip › Figure 4-source data 2/Figure4B_original_gel.png]

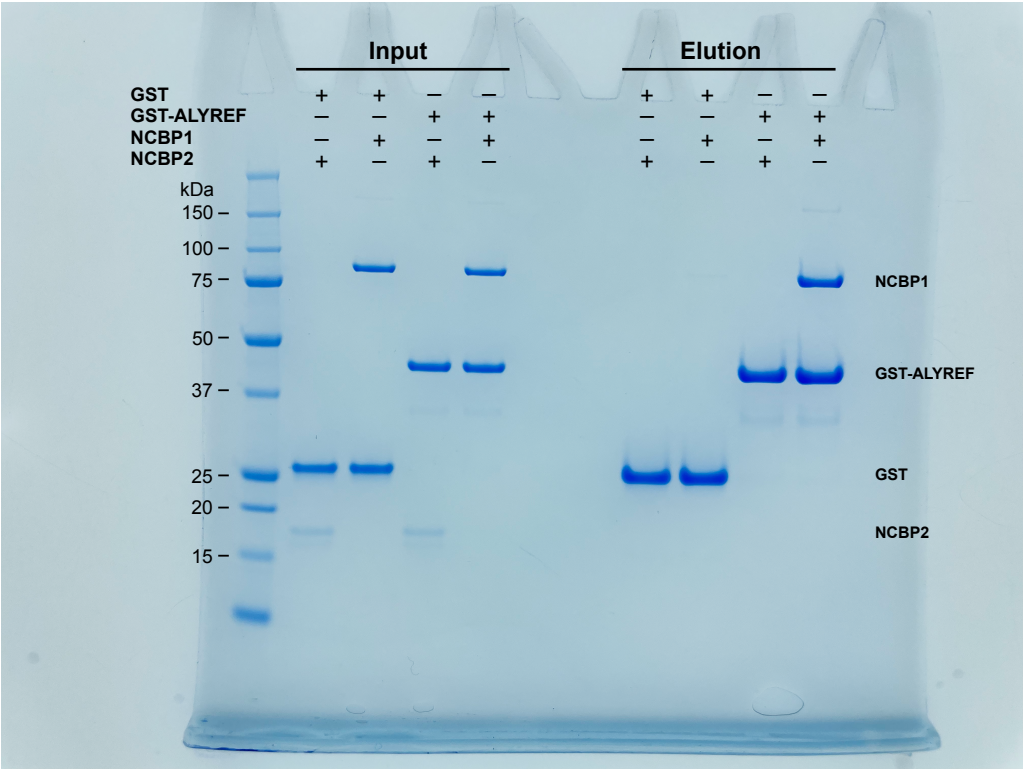

Supplement: Figure 4—source data 2. [file elife-91432-fig4-data2.zip › Figure 4-source data 2/Figure4B_gel_with_label.pdf]
